# Inducing G2/M Cell Cycle Arrest and Apoptosis through Generation Reactive Oxygen Species (ROS)-Mediated Mitochondria Pathway in HT-29 Cells by Dentatin (DEN) and Dentatin Incorporated in Hydroxypropyl-β-Cyclodextrin (DEN-HPβCD)

**DOI:** 10.3390/ijms17101653

**Published:** 2016-10-18

**Authors:** Al-Abboodi Shakir Ashwaq, Mothanna Sadiq Al-Qubaisi, Abdullah Rasedee, Ahmad Bustamam Abdul, Yun Hin Taufiq-Yap, Swee Keong Yeap

**Affiliations:** 1 MAKNA-UPM, Cancer Research Laboratory, Institute of Bioscience, University Putra Malaysia, 43400 Selangor, Malaysia; mothanna-alqubaisi@yahoo.com (M.S.A.-Q.); zer2crystals@gmail.com(A.B.A.); 2 Department of Veterinary Laboratory Diagnosis, Faculty of Veterinary Medicine, University Putra Malaysia, 43400 Selangor, Malaysia; rasedee@gmail.com; 3 Department of Biomedical Science, Faculty of Medicine & Health Science, University Putra Malaysia, 43400 Selangor, Malaysia; 4 Department of Chemistry, Faculty of Science, University Putra Malaysia, 43400 Selangor, Malaysia; taufiq@upm.edu.my; 5 LIVES, Institute of Bioscience, University Putra Malaysia, 43400 Selangor, Malaysia; skyeap2005@gmail.com

**Keywords:** Dentatin, cytotoxicity, apoptosis, cytostatic, reactive oxygen species (ROS)

## Abstract

Dentatin (DEN), purified from the roots of *Clausena excavata* Burm f., has poor aqueous solubility that reduces its therapeutic application. The aim of this study was to assess the effects of DEN-HPβCD (hydroxypropyl-β-cyclodextrin) complex as an anticancer agent in HT29 cancer cell line and compare with a crystal DEN in dimethyl sulfoxide (DMSO). The exposure of the cancer cells to DEN or DEN-HPβCD complex leads to cell growth inhibition as determined by MTT (3-(4,5-dimethylthiazol-2-yl)-2,5-diphenyltetrazolium bromide) assay. To analyze the mechanism, in which DEN or DEN-HPβCD complex causes the death in human colon HT29 cancer cells, was evaluated by the enzyme-linked immunosorbent assay (ELIZA)-based assays for caspase-3, 8, 9, and reactive oxygen species (ROS). The findings showed that an anti-proliferative effect of DEN or DEN-HPβCD complex were via cell cycle arrest at the G2/M phase and eventually induced apoptosis through both mitochondrial and extrinsic pathways. The down-regulation of poly(ADP-ribose) polymerase (PARP) which leaded to apoptosis upon treatment, was investigated by Western-blotting. Hence, complexation between DEN and HPβCD did not diminish or eliminate the effective properties of DEN as anticancer agent. Therefore, it would be possible to resolve the conventional and current issues associated with the development and commercialization of antineoplastic agents in the future.

## 1. Introduction

Cancer is a devastating disease that wreaks havoc on one’s life. As stated by the World Health Organization (WHO) report, there were 7.9 million deaths in 2007 because of this disease, and it is predicted that this number will grow up to 18 million in 2020 [1]. Specifically, colorectal cancer is the fourth most common malignant disease in the United States [2]. Chronic and metastatic diseases and their treatments continue to be the focus of clinical attention. While there are huge efforts carried out by most recent researchers to elucidate new molecular target-based molecules, there are also growing interests about natural substances are being considered for application in chemotherapeutic procedures. For instance, phytochemicals, named polyphenols, extracted from different plants parts, such as fruits, leaves, roots, have powerful anticancer effects [3]. Using in vitro cytotoxicity assays for these phytochemical compounds against cancer cells, the mechanisms by which these compounds inhibit cancer growth can be explored. It has been widely revealed that the inhibition mechanism by which these natural compounds work includes inhibition of tumour cell mediated protease activity, promotion of apoptosis and induction of cell cycle arrest [4].

Dentatin (DEN) (5-methoxyl-2,2-dimethyl-10-(1,1-dimethyl-2propenyl) dipyran-2-one), usually purified from *Clausena excavata*, is one of the vital coumarins which are categorized under polyphenols it is. It has been verified that DEN induces apoptosis in human prostate cancer through inhibition of nuclear factor-κB (NF-κB) nuclear translocation and downregulation of the anti-apoptotic molecules such as Bcl-2, Bcl-xL, and Survivin [5]. In addition, using the mitochondrial signaling pathway, DEN has also stimulated apoptosis in human breast cancer MCF-7 and human hepatocellular carcinoma HepG2 cells lines [6,7]. The drugs’ design and development have two critical factors that affect and limit their therapeutic application, represented by poor aqueous solubility and rate of dissolution [8]. The administration of poorly soluble drugs that belong to class II (high permeability, low solubility) or IV (low permeability, low solubility) of biopharmaceutical classification system (BCS), represents a major challenge [9]. Cyclodextrin (CD) complexation came into view and presented a great interest [10]. In 21st century, using CD represents a novel plan which has emerged to tackle such formulation problems, thus CD has increased the lipophilic compounds’ solubility and their dissolution rate [11].

Cyclodextrins are cyclic molecules composed of six, seven and eight (α, β, and γ-cyclodextrin) glucose units bonded with α-1,4 glycosidic linkages that mold into a truncated cone with a hydrophilic exterior and a hydrophobic cavity [12,13,14]. The pharmaceutical industry takes great interest in cyclodextrins (CDs) because of their biological nature. Also, the hydrophobic cavities of CDs enable selective binding of different inorganic, organic and biological molecules to form supramolecular complexes without any structural transformations [15,16,17]. This last point is the most significant property which draws the attention of pharmacologists toward CDs. To overcome the solubility limitations and safety of many compounds associated with innate CDs, several CDs have been synthesized and modified, which include hydroxypropyl and hydroxyethyl CDs [18], carboxymethyl CDs [19], methylated and alkylated cyclodextrins [20], sulfoalkyl ether CDs [21], and branched CDs [14,22]. Hydroxypropyl-β-cyclodextrin (HPβCD) is a characteristic hydroxypropyl CD, which is commonly used as a complex agent to improve the aqueous solubility of many drugs [23,24]. We used HPβCD to formulate an inclusion complex with DEN by freeze-drying technique.

The aim of this work was to study the cytotoxicity of both pure DEN dissolved in dimethyl sulfoxide (DMSO) alone and DEN incorporated into HPBCD cavity against human colon cancer HT29 cell line. Moreover, we determine the cell cycle arrest and the death pathway that the treated cells responded to using our compounds by cytometry analysis and quantifications of some apoptotic proteins, respectively. Eventually, it would be fruitful to move the compounds from poor aqueous solubility compounds to compounds dissolved in water without using organic solvent (which has toxic effects on the normal cells). Thereby, these findings will open the door for further investigation of this complex (DEN-HPβCD) via in vivo studies in the future.

## 2. Results

### 2.1. Cellular Sensitivity of Cells to DEN and DEN-HPβCD

**Figure 1:**
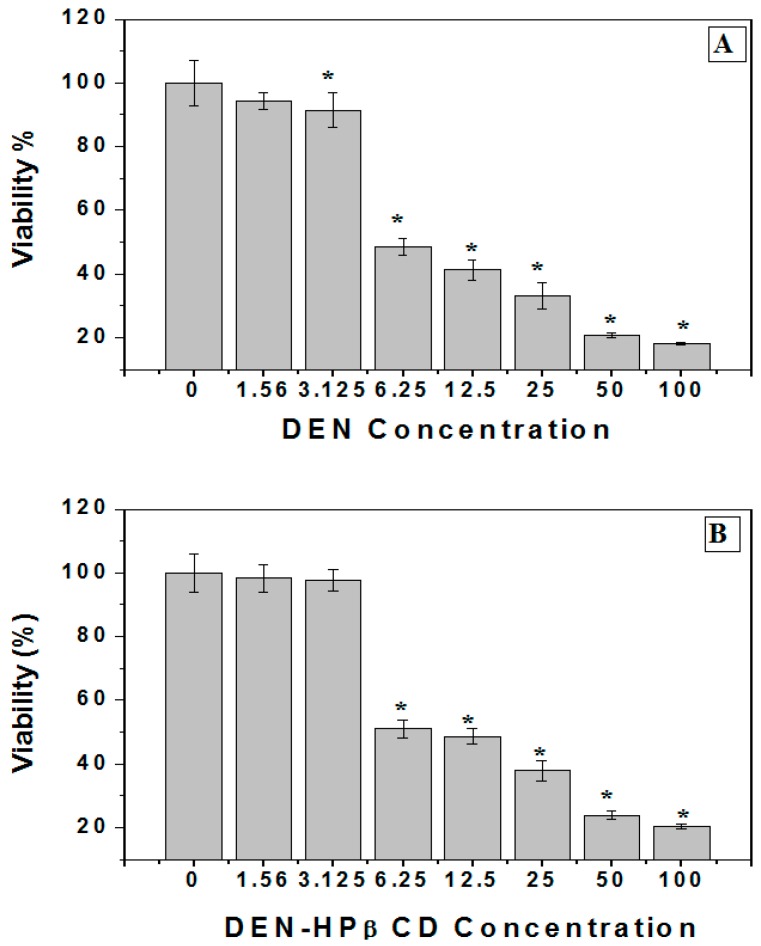
(**A**) Cytotoxicity of DEN (Dentatin) against human colon cancer cells (HT29). The cells were plated in 96-well plates and then exposed to 100, 50, 25, 6.25, 3.125 and 1.25 µg/mL of DEN for 72 h. The viability of treated cells were measured by using an MTT assay. Mean ± standard deviation (SD). (*n* = 3 well/treatment). * *p* < 0.05 compared with untreated cells; (**B**) Cytotoxicity of DEN-HPβCD (hydroxypropyl-β-cyclodextrin) complex against human colon cancer cells (HT29). The cells were plated in 96-well plates and then exposed to 100, 50, 25, 6.25, 3.125 and 1.25 µg/mL of DEN for 72 h. The viability of treated cells were measured by using an MTT assay. Mean ± standard deviation (SD). (*n* = 3 well/treatment). * *p* < 0.05 compared with untreated cells.

In this study, the MTT assay was used to assess the cytotoxicity activity against the human colon cancer cells (HT29) from both the dissolution of DEN in organic solvent DMSO and DEN incorporation in HPβCD complex. Figure 1A illustrates that DEN exhibited strikingly high cytotoxicity impact against human colon cancer cells when used in a dose-dependent manner, and with high concentration, these impacts were especially significant (*p* < 0.05). Consequently, the treated cells exhibited a gradual decline in viability (*p* < 0.05) in comparison to the untreated cells. The concentration of DEN that causes death of 50% of tested cells or the IC_50_ value of DEN was found to be 5.6 µg/mL. Earlier research performed by [7] revealed similar toxicity against Estrogen Receptor positive (ER+) MCF-7 where IC_50_ value was at 6.1 µg/mL. In the study mentioned above, DEN exhibited fewer side-effects on the normal cells in comparison to the cancer cells. The DEN-HPβCD complex also exhibited growth inhibition of treated cells with IC_50_ at 8.5 µg/mL, as shown in Figure 1B.

### 2.2. Morphological Examination of Treated Cells

**Figure 2:**
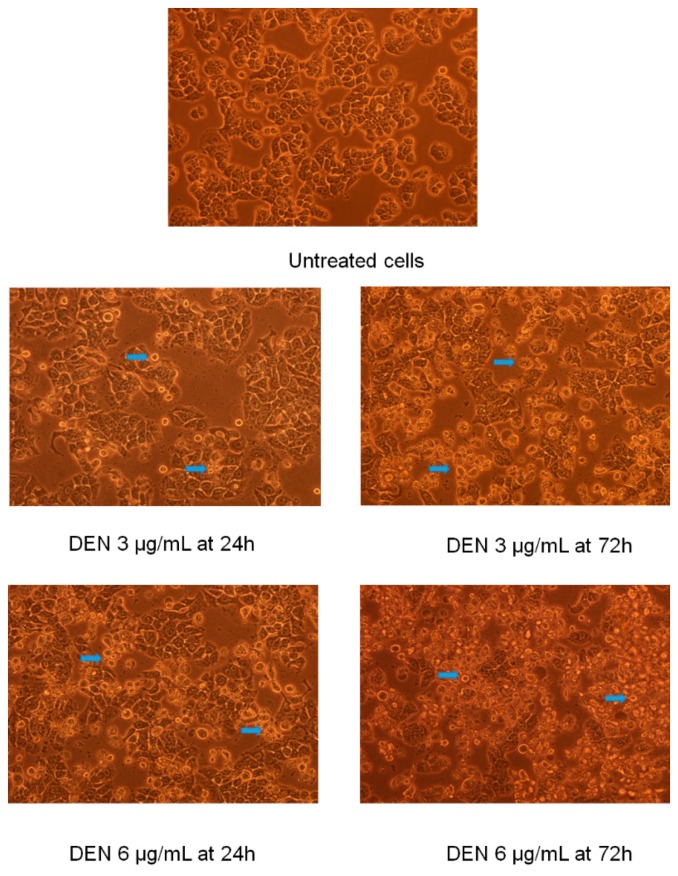
The morphological changes of HT29 treated cells with (3.125 and 6.25 µg/mL) of DEN dissolved in dimethyl sulfoxide (DMSO) for 24 and 72 h. Note: blue arrows indicate apoptotic cells (200×).

**Figure 3:**
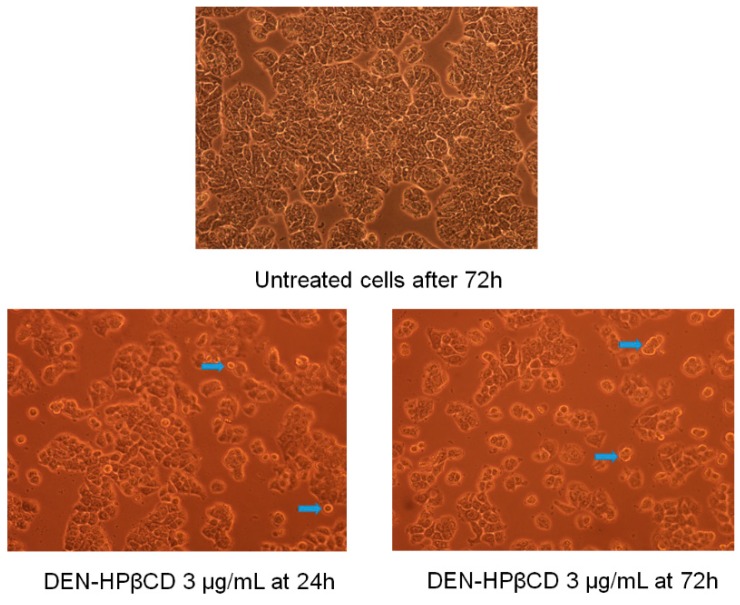

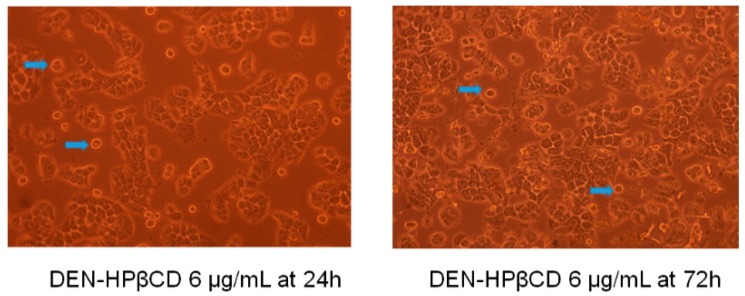
The morphological changes of HT29 treated cells with (3.125 and 6.25 µg/mL) of DEN-HPβCD complex for 24 and 72 h, Note: blue arrows indicate apoptotic cells (200×).

On examining the treated cells under inverted microscope, it was observed that there were remarkable alterations in the morphology of the cells and significant impacts on the physiology of the cells due to high influence of DEN and DEN-HPβCD. Moreover, with elevated dose and exposure time, this influence was growing. The morphological changes in the treated cells had various manifestations such as floating, detached, spherical, shrunken, and dispersed cells with cytoplasmic shrinkage and membrane blebbing. However, none of these changes were observed in the untreated cells; the cells exhibited healthy shape and adherence to the basic plates as shown in Figure 2. These alterations in morphology and physiology of the cells were accredited to the potential cytotoxicity impacts of DEN and its ability to induce cell death through apoptosis. The results of this experiment were similar to the conclusions of earlier research carried out by [6], where increase in the number of floating and spherical cells was observed after the cells were treated with DEN in a time-dependent manner. Although DEN-HPβCD treated cells also exhibited changes in morphology, it was slightly less compared to the alterations noticed in cells treated with DEN dissolved in DMSO (shown in Figure 3). Which attributed to accumulated compound in the complex, which then eventually got gradually released to the environment.

### 2.3. Trypan Blue Dye Exclusion

**Figure 4:**
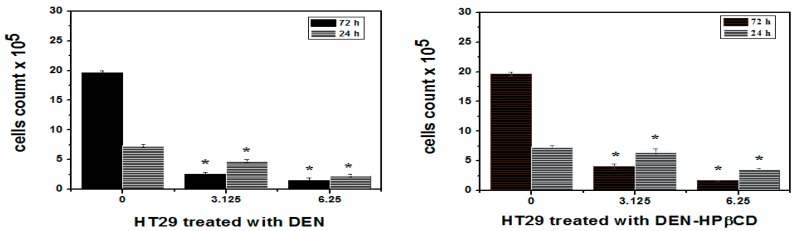
Trypan blue exclusion assay for cell viability of HT29 treated cells after 24 and 72 h of treatment with DEN and /or DEN-HPβCD complex. Mean ± standard deviation (SD). (*n* = 3 well/treatment). * *p* < 0.05 compared with untreated cells.

The cancer cells incubated for 24 and 72 h at two varying concentrations of 3.125 and 6.25 μg/mL were assessed to evaluate the anti-proliferative activity of DEN and DEN-HPβCD complex. The trypan blue dye exclusion technique was used to analyse the influence of the DEN and DEN-HPβCD complex on cell proliferation. As depicted in Figure 4, the HT29 cells exhibited more sensitivity towards the anti-proliferative effect of DEN than that of DEN-HPβCD complex. The number of viable treated cells had been decreased from (7.33 ± 0.3) × 10^5^ cells/mL to (4.76 ± 0.2) × 10^5^ and (2.33 ± 0.3) × 10^5^ cells/mL after being treated with 3.125 and 6.25 μg/mL frequently, for 24 h. 72 h of exposure to 3.125 and 6.25 μg/mL of DEN was sufficient to decrease viable treated cells to (2.63 ± 0.3) × 10^5^ and (1.56 ± 0.3) × 10^5^ cells/mL compared with untreated cells, which were (19.63 ± 0.3) × 10^5^ cells/mL. The HT29 cells exhibited lower sensitivity to DEN-HPβCD complex compare with DEN-treated cells. After 24 h, the count of viable HT29 cells treated with DEN-HPβCD complex at a concentrations of 3.125 and 6.25 μg/mL stood at (6.50 ± 0.5) × 10^5^ and (3. 53 ± 0.2) × 10^5^ cells/mL, while that for untreated cells was at (7.33 ± 0.3) × 10^5^ cells/mL. Furthermore, the viable HT29 cells declined steeply from (4.06 ± 0.4) × 10^5^ to (1.70 ± 0.09) × 10^5^ when exposed to DEN-HPβCD complex at a concentration of 3.125 and 6.25 μg/mL for 72 h which was significant compared with untreated cells, which were (19.63 ± 0.3) × 10^5^ μg/mL.

### 2.4. Determination of Apoptosis by Using AO/PI Double Staining

**Figure 5:**
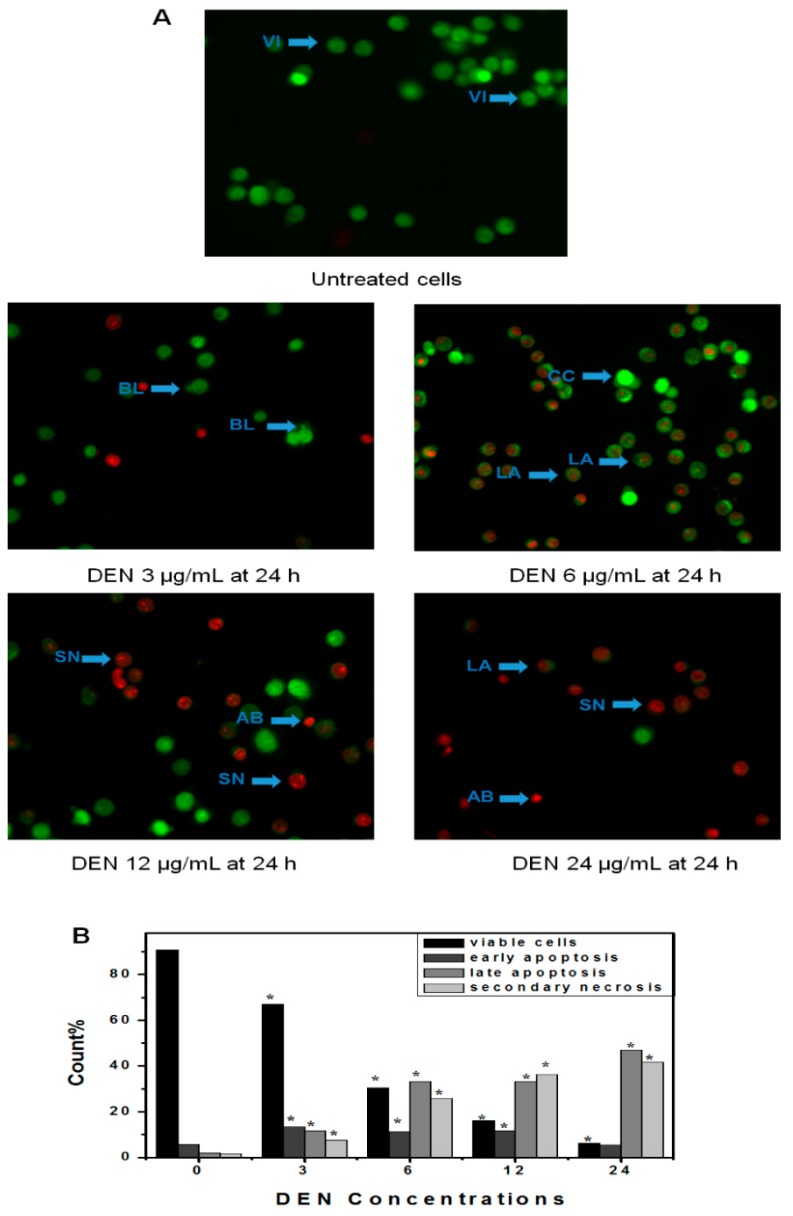
(**A**) Fluorescent micrographs of double-stained HT29 cells (acridine orange and propidium iodide). Untreated cells appeared normal structure without any features of apoptosis and necrosis. The early apoptosis features were seen after treatment with DEN (3 µg/mL) representing intercalated acridine orange (bright green) with fragmented DNA and Blebbing after 24 h. The hallmarks of late apoptosis which represented by orange colour were noticed after 24 h of treated with (6 µg/mL) which increased with increasing the concentration. Cells with bright red colour and big size representing secondary necrosis were visible after treated with higher concentration at 24 h. **VI**, viable cells; **BL**, blebbing of the cell membrane; **CC**, chromatin condensation; **LA**, late apoptosis; **SN**, secondary necrosis; **AB**, apoptotic bodies. Images are representative of one of the three similar experiment; (**B**) Count cells on different microphotographs. Note: the magnification is 40×. * *p* < 0.05 compared with untreated cells.

**Figure 6:**
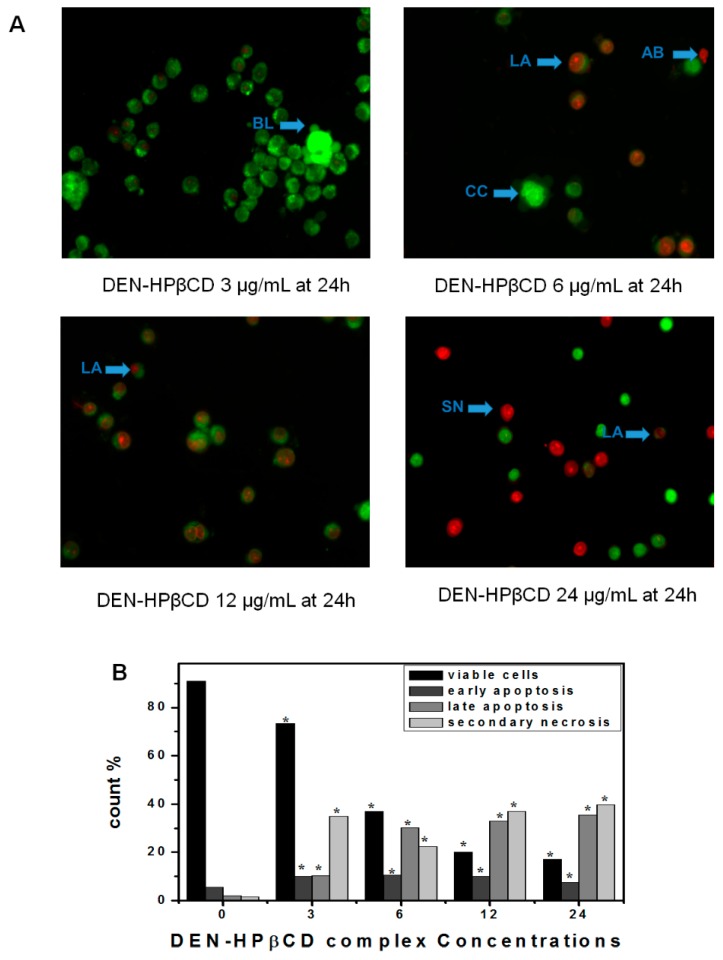
(**A**) Fluorescent micrographs of double-stained HT29 cells (acridine orange and propidium iodide). Untreated cells appeared normal structure without any features of apoptosis and necrosis. The early apoptosis features were seen after treatment with DEN-HPβCD complex (3 µg/mL) representing intercalated acridine orange (bright green) with fragmented DNA and Blebbing after 24 h. The hallmarks of late apoptosis which represented by orange colour were noticed after 24 h of treated with (6 µg/mL) which increased with increasing the concentration. Cells with bright red colour and big size representing secondary necrosis were visible after treated with higher concentration at 24 h. **VI**, viable cells; **BL**, blebbing of the cell membrane; **CC**, chromatin condensation; **LA**, late apoptosis; **SN**, secondary necrosis; **AB**, apoptotic bodies. Images are representative of one of the three similar experiment; (**B**) Count cells on different microphotographs. Note: the magnification is 40×. * *p* < 0.05 compared with untreated cells.

Cells treated with DEN and DEN-HPβCD complex were examined under inverted microscope (AxiO Vert.A1 FL-LED, Nikon Corporation, Tokyo, Japan), and it was observed after 24 h that there was moderate apoptosis in the form of nuclear chromatin condensation and blebbing in the cells. On the other hand, no noticeable apoptosis was found in the negative control group and the cells were seen to have undamaged green nuclei. In addition to this, early-stage apoptotic cells in the form of crescent-shaped or granular yellow-green acridine orange (AO) nuclear staining were observed in the experimental group (shown in Figure 5 and Figure 6). Within the cells, there was asymmetrical localization of staining. The number of early-stage apoptotic cells increased with an increase in the concentrations of DEN and DEN-HPβCD complex. At high concentrations of 12 µg/mL, late-stage apoptotic cells were observed, which had asymmetrically localized and concentrated orange nuclear propidium iodide (PI) staining, and which eventually converted to necrosis cells at a concentration of 24 µg/mL. An uneven orange-red fluorescence was observed at the periphery of necrotic cells as they expanded in volume. It appeared that the cells were undergoing the process of disintegration. The experiment results indicated that when the cells were treated with DEN and DEN-HPβCD complex in a dose-dependent manner, morphological changes related to apoptosis were induced in the cells.

### 2.5. Detection of Caspase-9, 3 and 8 Activity

**Figure 7:**
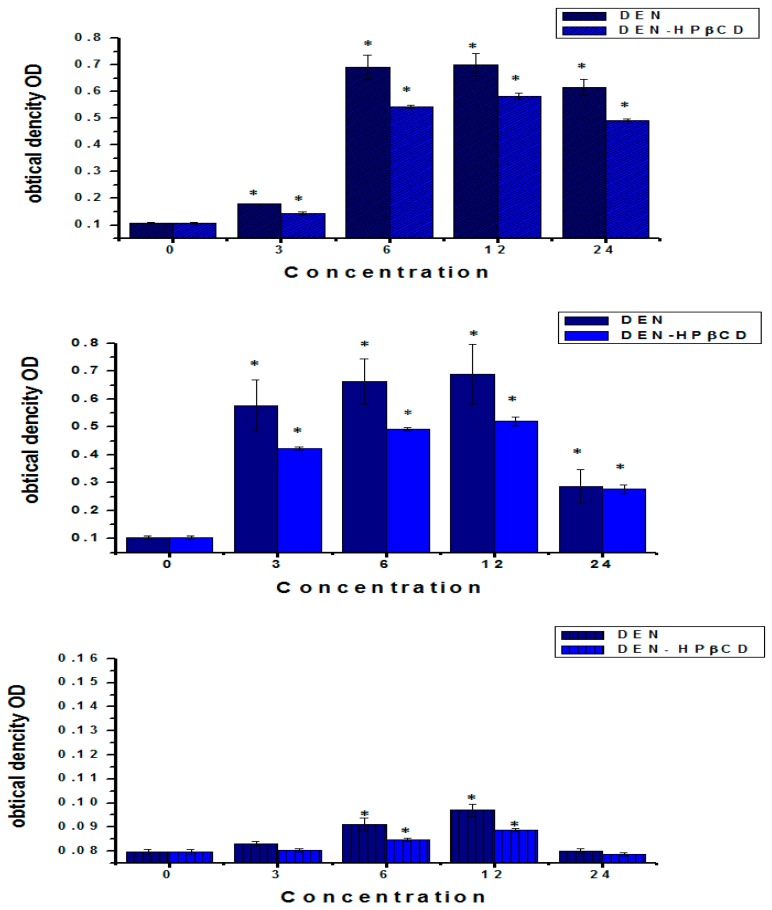

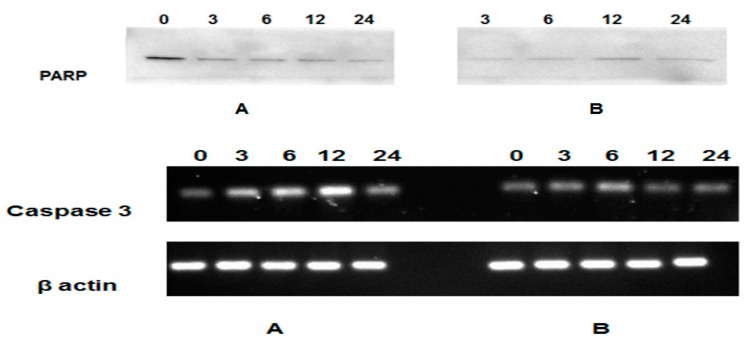
Treatment of HT29 cells with DEN and/or DEN-HPβCD complex showed in activation of caspase-3, 9 and 8. Cells were treated with 3, 6, 12 and 24 µg/mL for 24 h, and then determination of caspase-3, 9 and 8 activity was done. Mean ± standard deviation (SD). (*n* = 3 well/treatment). * *p* < 0.05 compared with untreated cells. RT-PCR analysis for caspase-3 mRNA expression was stimulated in treated and untreated cells. β-actin used as control. **Part A** represents caspase-3 and β actin mRNA expression in DEN-HT29 treated cells, **Part B** represents caspase-3 and β actin mRNA expression in DEN-HPβCD-HT29 treated cells. The effect of DEN and DEN-HPβCD complex on apoptosis regulatory proteins in the treated HT29 colon cancer cell line was determined by Western blot assay. Detection of poly(ADP-ribose) polymerase (PARP).

Caspase-3 is a significant executive enzymatic marker of apoptosis, while Caspase-9 and 8 are initiators for the mitochondria-mediated (intrinsic and extrinsic) apoptotic pathway. By using the Caspase-3/9 Colorimetric Assay Kit and Caspase-8 IETD-R110 Fluorometric and Colorimetric Assay Kit, the caspase-3, 9 and 8 activities of DEN and DEN-HPβCD complex-treated cells were assessed. Figure 7 depicts the considerable increase in activity of all caspases in the treated HT29 cells, compared to that of the untreated cells. The caspase-3, 9 and 8 activities appear to be almost parallel to the increase in doses of DEN and DEN-HPβCD complex. The HT29 cells that were treated with 12 μg/mL of DEN or DEN-HPβCD complex exhibited the highest caspases activity. Moreover, after 24 h, the caspase-3, 9 and 8 activity increased 6.9, 7 and 1.28 times more frequently with an increase in the dose of DEN (12 μg/mL) in the treated cells, compared to that of the untreated cells. On the contrary, the caspases activity declined at the highest concentration of 24 μg/mL. After 24 h of incubation of the cells treated with DEN-HPβCD complex, it was observed that the Caspase-3, 9 and 8 activity increased to about 5.8, 5.2 and 1.14 times than that in the untreated cells (shown in Figure 7), which again declined at a concentration of 24 µg/mL. 

### 2.6. Induction of Cell Cycle Arrest by DEN and DEN-HPβCD Complex

**Figure 8:**
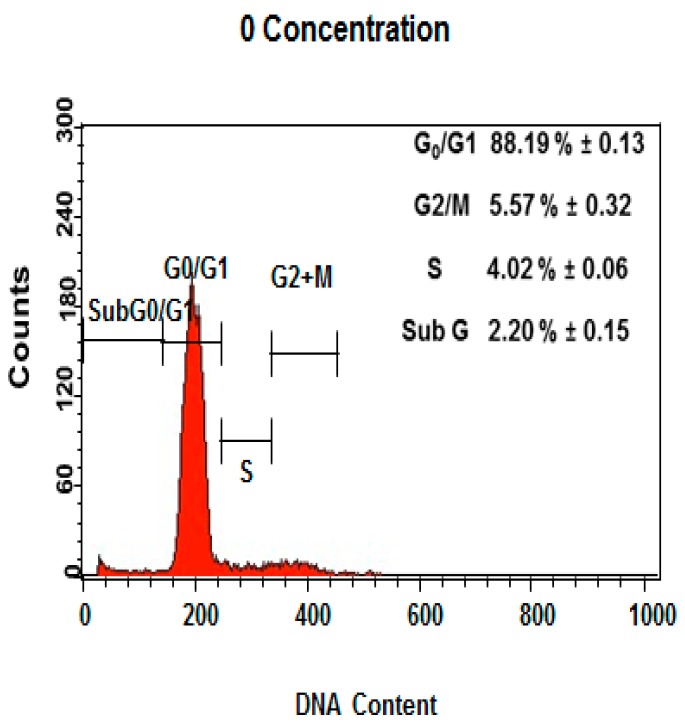

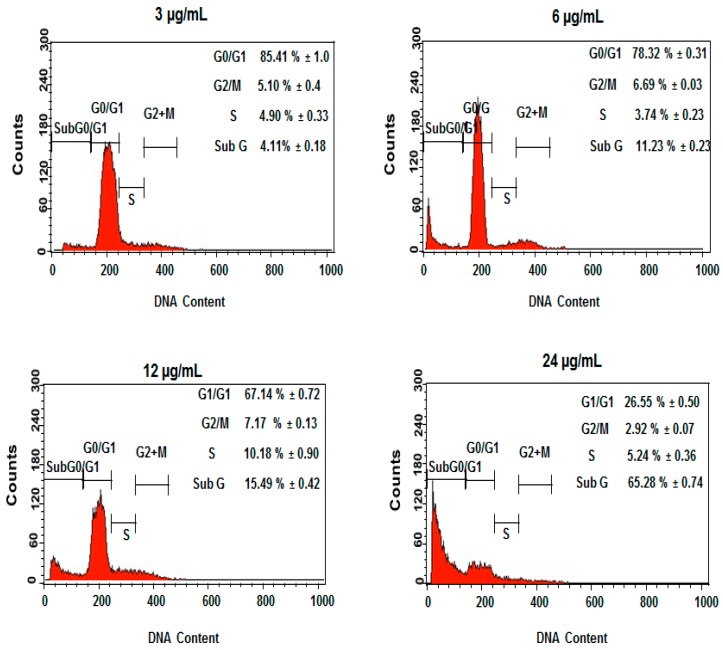
The concentrations (3, 6, 12 and 24 µg/mL) of DEN in HT29-treated cells included G2/M cell cycle arrest. Results are represented one of the three independent experiments.

**Figure 9:**
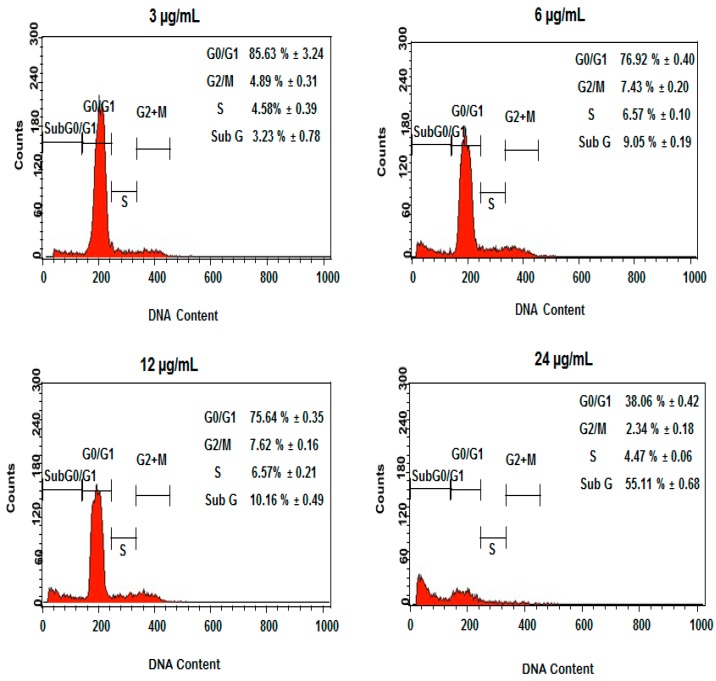
The concentrations (3, 6, 12 and 24 µg/ml) of DEN-HPβCD complex in HT29-treated cells included G2/M cell cycle arrest. Results are represented one of the three independent experiments.

Further analysis of the cell cycle progression in DEN and DEN-HPβCD complex-treated cells was carried out through flow cytometry. The experiment results indicate that DEN and DEN-HPβCD complex induced cell cycle in the treated HT29 cells at the G2/M phase. Figure 8 shows that there was a considerable dose-dependent accumulation of cells in the G2/M phase, accounting for 6.69% ± 0.03% and 7.17% ± 0.13% for DEN, respectively, after the cells were treated with 6 and 12 µg/mL concentration of DEN for 24 h. At the same time, DEN triggered a corresponding decrease in cells at G0/G1 phase and a considerably higher population of hypodiploid (sub G1/apoptosis). Thus, our results indicate that a blockage occurred at the G2/M phase, causing cell growth inhibition. Furthermore, the sub G peaks were further corroborated by the corresponding externalization of phosphatidylserine, indicating that apoptosis was interceded by cellular mitosis inhibition. In addition, as shown in Figure 9, the DEN-HPβCD complex appeared more similar activity for DEN in treated cells by inducing cell cycle arrest in G2/M phase. The ratio of accumulation cells was 7.43% ± 0.2% and 7.62% ± 0.1% after treated with 6 and 12 µg/mL frequently. Furthermore, the sub G peak was increased with increasing concentration to be 55.11% ± 0.6% after exposure to 24 µg/mL at 24 h.

### 2.7. Reactive Oxygen Species (ROS)

**Figure 10:**
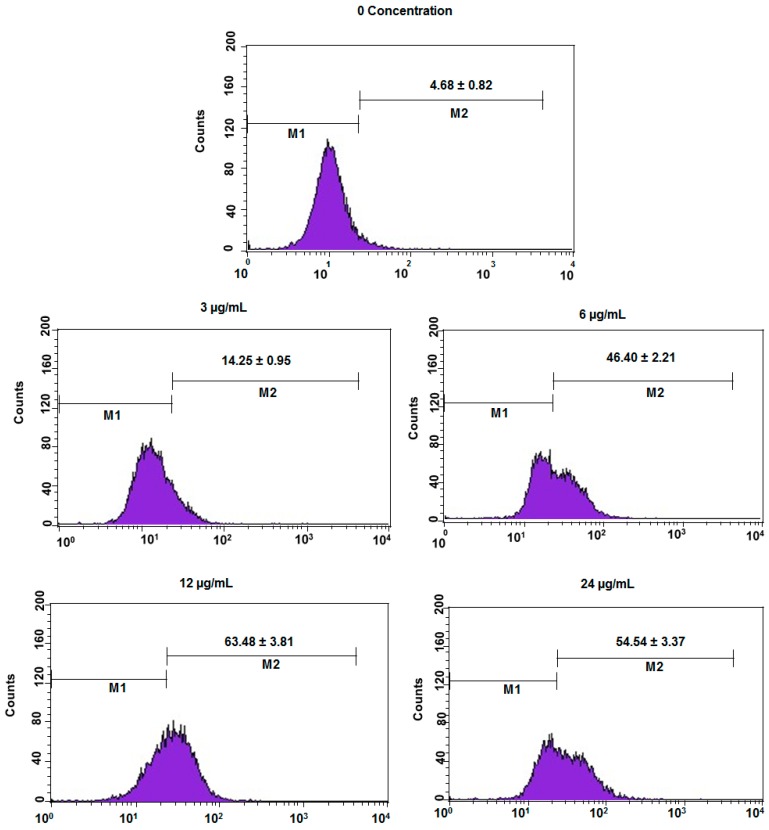
The concentrations (3, 6, 12 and 24 µg/mL) of DEN in HT29-treated cells induced ROS generation. Results are represented one of the three independent experiments. Note: M1 represented the cells that not producing ROS, M2 represented the cells that actively producing ROS.

**Figure 11:**
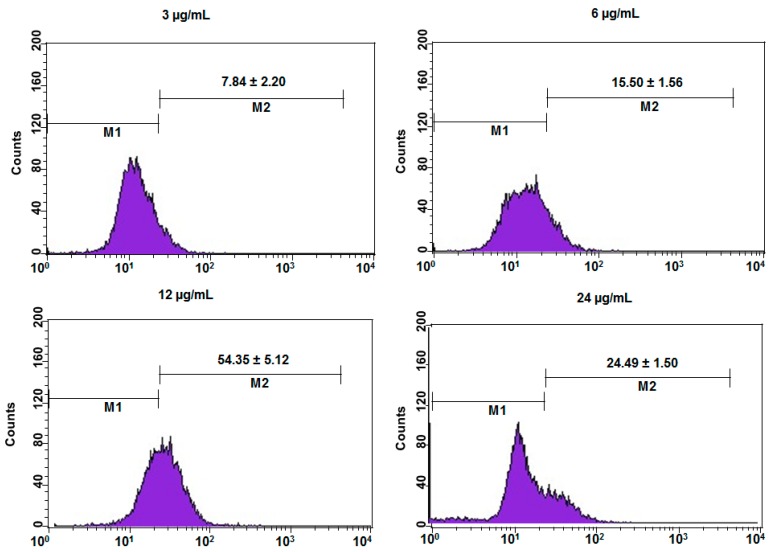
The concentrations (3, 6, 12 and 24 µg/mL) of DEN-HPβCD complex in HT29-treated cells induced ROS generation. Results are represented one of the three independent experiments. Note: M1 represented the cells that not producing ROS, M2 represented the cells that actively producing ROS.

Dichlorofluorescein fluorescence was used to find intracellular mitochondrial reactive oxygen species (ROS) generation in DEN or DEN-HPβCD complex treated cells. The presence and growth of the reactive oxygen species (ROS) in the HT29 cells treated with 3, 6, 12 and 24 μg/mL of DEN or DEN-HPβCD complex was considerably greater than that in the untreated cells (shown in Figure 10 and Figure 11). As depicted in Figure 10, with increasing concentration of DEN (3, 6 and 12 µg/mL), there was a steep increase in ROS (14.25, 46.40, and 63.48), while in the untreated cells, the growth of ROS was significantly slow (4.68). On the other hand, with the highest concentration of DEN (24 µg/mL), the level of ROS reduced to 53.45, which is less compared to the ROS level in DEN-treated cells (12 µg/mL). Figure 11 presented that the DEN–HPβCD complex induced significant growth of ROS by 11.6-fold (approximately) in HT29 treated cells after the cells were exposed to 12 µg/mL at 24 h, compared to untreated cells. However, this increasing was low compare to the increasing observed in DEN treated cells.

### 2.8. Expression of PARP 

The expression of PARP in the HT29 cells treated with or without DEN or DEN-HPβCD complex was tested by Western blot analysis. As shown in Figure 7, after 24 h treatment with 3, 6, 12 and 24 μg/mL of DEN or DEN-HPβCD complex, the levels of PARP protein have decreased significantly for all treated samples compared with the untreated cells.

## 3. Discussion

One of the most interesting area, where the oncologists work recently, is indicating the death pathway that cancer cells behaved following exposure to certain molecule; apoptosis vs. necrosis. The associated genes with apoptosis, which responded by up-regulating or down-regulating, are commonly screened. Secondary metabolites of many plant have showed anticancer effect against cancer cells through apoptosis. Apoptosis can take place directly or indirectly through the modulation of the course of tumor development via different cellular pathways [25,26]. Recently, the chemotherapy agents for recurrent and metastasis diseases have been developed. But, none of chemotherapy agents is safe even-though they are effective, and able to tackle the disease [27]. In addition, absence of selectivity and short blood circulation time of chemotherapy agents, which in turn, will damage not only cancer cells but also normal and healthy tissue [28]. Thus, there is importunate need to implement novel technology based on combination strategy of cyclodextrin complexation with active compounds to make them more useful and acceptable therapeutic agents. Furthermore, this combination strategy has been provided with some advantages for drug delivery systems such as improvement/enhancement of drug solubility and stability, increasing of its bioavailability and dissolution, reducing of its toxicity, and modifying of its physicochemical characteristics [29].

As a result of its chemical properties (like most coumarins), DEN have biomedical applications as anticancer agent [30]. These results focused on the effect of DEN and DEN-HPβCD complex against HT29 cancer cell line. It can be considered as the first documentation on DEN and DEN-HPβCD complex. DEN has induced apoptotic in HT29 cancer cell line through increased ROS, mitochondria-dependent activation of caspase cascade, and G2/M cell cycle arrest.

In vitro, cytotoxicity of both DEN and DEN-HPβCD should be screened before testing in animal studies [31,32]. Cytotoxicity determinations include concentration response. This is important because it gives a preclinical evaluation of the range where toxicant-induced cytotoxicity or response takes place [33]. Figure 1A,B showed how HT29 cells responded differently following exposure to DEN and DEN-HPβCD complex. Based on the viability values, it can be said that HT29 cells showed more sensitivity to the DEN dissolved in DMSO than the DEN-HPβCD complex after 72 h of exposure. This difference can be attributed to DMSO’s role and the time needed for drug release. Therefore, exposure time may be prolonged by gradually releasing the DEN from the complex [34]. Moreover, higher resistance was displayed by normal breast MCF10a cells as previously mentioned by [7]. To confirm the selectivity, it can compare the cytotoxicity effects of DEN and DEN-HPβCD complex for cancer cell line with normal cell lines. This is an important part when the evaluation for a newly formulated drug is needed [35]. It was observed that cells treated with DEN and DEN-HPβCD complex exhibited morphological changes closely associated with apoptosis (programmed cell death). Under a light microscope, the morphological characteristics of apoptosis, as a result to treatments with the DEN and DEN-HPβCD complex, are revealed. The alterations referred to early and late phases of apoptotic mechanism in the cells treated with the DEN and DEN-HPβCD complex were demonstrated using the AO/PI double staining fluorescent assay. Under inverted microscope, the dose-dependent increases in apoptotic population were observed. 

The pathways of apoptosis involve an energy-dependent cascade of molecular events, and are considered to be extremely sophisticated [36]. Apoptosis can take place via two pathways. The first pathway is widely known as the intrinsic or the mitochondrial pathway. The second pathway is the extrinsic or the death-receptor dependent pathway [37]. Free radical generation during cytotoxicity results in the liberation of reactive oxygen species (ROS). Reports reveal that increased ROS levels could cause oxidative damage to cells. ROS is generally localized in the mitochondria and it is important in activating apoptosis and thus inducing cell death [38]. In other words, ROS plays an important role in apoptosis induction by activating the mitochondria intrinsic pathway [7]. Current study revealed that there are increasing in the reactive oxygen species levels in DEN and DEN-HPβCD complex-treated HT-29 cells compared with untreated cells in dose dependent manner. In contrast, the efficacy of the reactive oxygen species decreased when treated cells with highest concentration 24 µg/mL. The results above reveal that DEN and DEN-HPβCD complex are very effective in inducing ROS in treated cells. Hence, the HPβCD didn’t effect on the DEN activity in inducing ROS. An increase in ROS in treated cells was also associated with the regulation of both anti-apoptotic protein (Bcl-2) and glutathione (GSH) [39]. The activation of cysteine proteases, which cleaves numerous specific cellular substrates, greatly affects the intrinsic pathway of apoptosis. An increase in the oxidative stress in the mitochondria resulted into a leakage of cytochrome C from the mitochondrial inter-membrane space to the cytoplasm. This is connected with Apf-1 and caspase 9 and resulted in the formation of an apoptosome complex. Consequently, the executive protein caspase 3 was activated by this apoptosome and cell death was subsequently triggered [39]. Our results revealed that exposure to DEN and DEN-HPβCD complex has significantly increased the levels of caspase 9 and 3 compared with the untreated cancer cells. Based on this result, it can be concluded that apoptosis was induced by the DEN and DEN-HPβCD complex via the intrinsic mitochondria pathway. This result supports previous studies on the role of DEN in up-regulating of caspase 9 and 3 genes in prostate cancer cells [5,6]. Moreover, inducing caspase 8 using DEN and DEN-HPβCD complex in treated cells can indicated the extrinsic pathway of apoptosis is also behaved. Caspase-8 works in two sides; the first one directly activates the downstream effector caspase-3, the second, caspase-8 induces the truncated Bid (tBid), which in turn results in the release of cytochrome C from the mitochondria into the cytosol. All these steps result in the activation of caspase-9 and caspase-3 [40]. The highest concentration of DEN (24 µg/mL) also resulted in decreased caspase 9, 8 and 3 levels. This result supports the idea that caspase-mediated apoptosis takes place at low concentrations and, at higher concentrations, treated cells undergo secondary apoptosis associated with proteolysis [41].

With flow cytometry cell cycle analysis revealed that the apoptosis was induced in treated cell after exposure to DEN or DEN-HPβCD complex for (24 h). That can been seen obviously by increasing cells population at the sub-G0 phase. This increasing in Sub-G0 portion was concomitant with the observed increase of G2/M cell ratio, which can also be attributed to the efficacy of the DEN or DEN-HPβCD complex in regulating p53 [42]. That play vital role in sequesters cyclin B1-Cdc2 complex by inducing p21 in response to the DNA damage that took place after treatment [43]. In turn, p53 can induce apoptosis by up-regulating of various of apoptosis-associated genes [42,44].

For further investigation and validation of enzyme-linked immunosorbent assay ELIZA and cytometry findings, western blot assay was done to measure expression levels of PARP protein. The fragmentation of DNA was induced by extrinsic and intrinsic pathway, at this time the cell tries to prevent and repair with the help of poly(ADP-ribose) polymerase (PARP) [45]. The preventing process of DNA fragmentation that the cell attempts to do were inhibited by treatment with DEN or DEN-HPβCD complex that in turn down regulated PARP and making it unable to function properly. Moreover, Molecular mechanism by Reverse Transcription-Polymerase Chain Reaction (RT-PCR) Analysis showed increasing in the expression of caspase-3 after exposure to DEN or DEN-HPβCD complex. 

However, there is poor knowledge about physiological effects of our formulation on human body if applied as real medication. The route of administration and the mechanism of elimination of this carrier are entirely unknown. Because of that, this study offer additional avenues to exploit this complex in vivo models studies for future investigations.

## 4. Materials and Methods

### 4.1. Materials

For this experiment, Dulbecco’s modified Eagle’s medium (DMEM), 3-(4,5-dimethylthiazol-2-yl)-2,5 diphenyltetrazolium bromide (MTT), dimethyl sulfoxide (DMSO), Trypsin, trypan blue dye, phosphate-buffered saline (PBS), and were purchased from Sigma Chemical Company (St. Louis, MO, USA). The HPβCD (purity ≥ 98%) used in this investigation was procured from Sigma Aldrich (Taufkirchen, Germany). All the chemical materials and reagents and used were analytical grade, and ultrapure water was used during all the experimental steps.

### 4.2. Cell Culture

Human colon cancer HT29 cells bought from ATCC, Rockville, MD, USA and grown in Dulbecco’s Modified Eagles medium (DMEM) enhanced with 1% amphotericin B, 1% penicillin-streptomycin and 10% fetal bovine serum to grow the cells. A humidified incubator at a temperature of 37 °C and with 5% CO_2_ was used to incubate the flasks that contained cancer cells.

### 4.3. Preparation of the Inclusion Complex

Freeze-drying technique was utilized to prepare the DEN-HPβCD complex. This required preparation of 1:1 molar ratio DEN solutions in HPβCD 0.3264 g DEN was dissolved in 5 mL chloroform and mixed with 1.4 g HPβCD that was diluted in 20 mL of ultra-pure water. After stirring the mixture for 72 h at room temperature, the mixture was filtered using 0.45 m filter paper. The filtered clear solution was frozen at −80 °C, and then freeze-dried for 24 h at −55 °C.

### 4.4. Cytotoxicity MTT Assay 

In a 96-well tissue culture plate, the HT29 cells line were plated at 2 × 10^3^ cells/well by adding 200 μL of a 1 × 10^4^ cells/mL suspension to each well. To ensure attachment at 30% to 40% confluency, the plates were incubated for sufficient amount of time. The already present media was removed and fresh media (200 μL) was added containing DEN or DEN-HPβCD complex of different concentrations (1.56–100 μg/ mL), while the last row was left untreated. For 72 h, the plates were incubated at a temperature of 37 °C and 5% of CO_2_. On completion of the incubation with the compounds, the media was removed and the cells were washed three times with PBS buffer so that the cells did not have any trace of DEN or DEN-HPβCD complex, and then a fresh media was added. To every well, the MTT solution (20 μL) with a total volume of 200 μL was added and mixed gradually with the media, and later incubated at 37 °C with 5% CO_2_ for 4 to 6 h. Subsequently, the MTT-containing medium was aspirated off and gently replaced with DMSO (200 μL per well) so that the formazin crystals could be dissolved. The plates were read at 570 nm in a microtiter plate reader. Using the dose-response curves of each compound, the concentration of drug required to stop the cell growth by 50% (IC_50_) was determined.

### 4.5. Microscopic Examination of Cell Morphology

In the six-well plates, the HT29 cells (1 × 10^5^ cells/well) were seeded and incubated overnight so that the cells hold on to the plates. After this, the cells were exposed to 3.125 and 6.25 µg/mL of DEN and DEN-HPβCD complex for 24 and 72 h, respectively. A light-inverted microscope was then used to observe the changes in membrane and normal morphology of the cells.

### 4.6. Trypan Blue Exclusion Assay

To determine the antiproliferative effect of DEN or DEN-HPβCD complex, at first, the HT29 cells were seeded (1 × 10^5^ cells/well in DMEM) in the six-well tissue culture plates. After 24 h of incubation that enabled the cells to attach to the plates, the cells undergoing exponential growth were exposed to DEN or DEN-HPβCD complex at concentrations of 3.125 and 6.25 µg/mL. Then, for 24 to 72 h, the plates were incubated at a temperature of 37 °C, and CO_2_ presence of 5%. On completion of the incubation, the medium was removed, and the plates were cleaned with cold phosphate-buffered saline solution to remove the dead cells and replenished with a fresh medium of 1 mL of 0.05% (2 mg/mL) trypsin-ethylene diamine tetra acetic acid. Then, for 10 to 15 min, the plates were incubated at a temperature of 37 °C, till most of the cells confirmed as microscopically detached. The cells were then collected through centrifugation of the cell suspension at 1000 rpm for 10 min and discarding the supernatant. The resultant cell suspension (20 μL) was then blended with 20 μL of 0.4% trypan blue solution which led to the resuspension of the cells. Thereafter, by using a hemocytometer chamber, the dye-excluding viable cells were microscopically counted.

### 4.7. Morphological Evaluation of Apoptotic Cells by Acridine Orange (AO) Propidium Iodide (PI) Double Staining

To further confirmation role of DEN and DEN-HPβCD complex in processing cells death, acridine orange (AO) and propidium iodide (PI) double-staining were used, where the cells were examined under the (inverted microscope AxiO Vert.A1 FL-LED, Nikon Corporation, Tokyo, Japan). The treatment was carried out in the six-well tissue culture plates. The plating of the cells was done at (1 × 10^5^ cells/well in DMEM) and then the plates were treated with varying concentrations of DEN and DEN-HPβCD complex (3, 6, 12, and 24 μg/mL) in a dose-dependent manner for 24 h. The cells then underwent centrifugation at 300g for 10 min. The supernatant of the cell suspension was discarded and the cells were washed twice with PBS, so that the remaining media could be removed. Ten microliters of fluorescent dyes containing AO (10 mg/mL) and PI (10 mg/mL) in equal volumes were supplemented into the cellular pellet, and the freshly stained cell suspension was released into a glass slide and shielded with a cover slip. Under (inverted microscope AxiO Vert.A1 FL-LED), the slides were examined for 30 min till the fluorescence eventually faded. When AO and PI have characteristic to bind with DNA. Out of PI and AO, only AO has the potential to cross the plasma membrane of early and sustainable apoptotic cells. The identification criteria include: (i) viable or sustainable cells that show the presence of green nuclei with undamaged structure; (ii) a bright-green nucleus exhibiting chromatin condensation in the nucleus that reveal early apoptosis; (iii) dense orange spaces of chromatin condensation in the nucleus that exhibit late apoptosis and (iv) undamaged orange nucleus indicating secondary necrosis [5]. 

### 4.8. Caspase-3 and -9 Activity

By using the Caspase-3/9 Colorimetric Assay Kit (GenScript, Piscataway Township, NJ, USA) on the HT29 cells that were treated with varying concentrations of DEN or DEN-HPβCD complex (3, 6, 12, and 24 μg/mL) in a dose-dependent manner for 24 h, the extent of caspase-3, 9 activation in the cells was evaluated and measured. The cells were plated at a density of 1 × 10^6^ cell/well as per the manufacturer’s protocol (GenScript). The plated cells were treated with DEN or DEN-HPβCD, and the cell suspension was centrifuged. The cells were then collected and washed with PBS, lysed in 50 μL of cold lysis buffer and left on ice with vortex for 1 h. Thereafter, 50 μL of supernatant of each sample was mixed with 50 μL of 2× reaction buffer and 5 μL (200 μM) of DEVD-pNA substrate (caspase-3)/LEHD-pNA substrate (caspase-9), and the solution was incubated away from light for 4 h at a temperature of 37 °C. Then, the plates were studied at 405 nm.

### 4.9. Caspase-8 Activity 

By using the Caspase-8 IETD-R110 Fluorometric and Colorimetric Assay Kit (Biotium, Fremont, CA, USA) on the HT29 cells that were treated with different concentrations of DEN or DEN-HPβCD (3, 6, 12, and 24 μg/mL) in a dose-dependent manner for 24 h, the extent of caspase-8 activation in the cells was evaluated and measured. The plating of the cells was done at a density of 1 × 10^6^ cells/well. Once the cells were treated with DEN or DEN-HPβCD complex (3, 6, 12, and 24 μg/mL) in a dose-dependent manner for 24 h, the cells were collected through the process of centrifugation. The harvested cells or pellets were washed with PBS, lysed in 50 μL of chilled cell lyses buffer, and set down on ice for 10 min. At a temperature of 4 °C, the lysate was centrifuged for 5 min. Then, 50 μL of Assay Buffer was added to the centrifuged supernatant for every sample and blended well, and 5 μL of 1 mM Enzyme Substrate was further added to the samples. The samples were then made to undergo incubation at a temperature of 37 °C for 30 to 60 min. At 520 nm emission and 470 nm excitation, the plates were then readed.

### 4.10. Cell Cycle Analysis

With the use of flow cytometry analysis, the proportion of HT29 cancer cells in the different phases was assessed. The HT29 cells were seeded in the six-well plates at a density of (1 × 10^6^ cells/well) for 24 h, in accordance to the protocol defined by the manufacturer (BD Cycletest plus DNA Kit, BD Biosciences, San Jose, CA, USA). Then the cells were treated with 3, 6, 12 and 24 μg/mL of DEN or DEN-HPβCD complex, and incubated for 24 h. After harvesting the cells, 1 mL of buffer solution was added to the cells to create a resuspension, which was then centrifuged. Each tube was filled with 250 µL of solution a (trypsin buffer) and incubated at room temperature for 10 min. The tubed were then supplemented with 200 µL of solution B (trypsin inhibitor and RNase buffer) and incubated at room temperature for 10 min. 200 µL of cold solution C (PI stain solution) was further added to every tube and incubated using ice for 10 min in the dark. Then using the flow cytometry analysis (FACS Calibur), the cells were studied under 488 nm excitation, while the PI fluorescence was measured under 620–640 nm excitation (Becton-Dickinson, Franklin Lakes, NJ, USA).

### 4.11. Measurement of Reactive Oxygen Species (ROS) Level

By utilizing the total reactive oxygen species (ROS) assay kit (Ebioscience, San Diego, CA, USA). The production of intracellular reactive oxygen species (ROS) was ascertained. The experiment was carried out by following the instructions of the manufacturer. In six-well plates, the cells were seeded at (1 × 10^6^ cells/well) and then incubated for 24 h. After incubation, the cells were treated with varying concentrations of DEN or DEN-HPβCD complex for 24 h. Then, by using trypsin, the cells were detached. The detached cells were washed with PBS. Thereafter, the cells were treated with 100 µL ROS assay stain solution and incubated for 60 min at a temperature of 37 °C and CO_2_ presence of 5%. The BD face flow cytometer was then used for further analysis.

### 4.12. Western Blotting Analysis

To determine protein expression, HT29 cells were plated at 1 × 10^6^ cells/well in 6-well plate. Then the cells were treated with different concentrations of DEN or DEN-HPβCD complex for 24 h. Cells were collected and lysed in lysis buffer (50 mM Tris–HCl pH 8.0, 120 mM NaCl, 0.5% NP-40, 1 mM phenylmethylsulfonyl fluoride (PMSF). 40 µg of the protein was resolved on 10% sodium dodecyl sulfate SDS-polyacrylamide gels. After electrophoresis, the proteins were transferred to polyvinylidene difluoride (PVDF) membranes (Millipore, Darmstadt, Germany). The membranes were blocked with 5% nonfat dry milk in TBS-T (0.05% Tween 20) for 1 h at room temperature. Membranes were incubated with appropriate primary mouse antibody overnight at 4 °C, followed by incubated with horseradish peroxidase (HRP)-conjugated secondary anti-mouse antibody for 1 h at room temperature. Protein-antibody complexes were detected with chemiluminescence (ECL System) and exposed by autoradiography (ChemiDoc MP imaging System/Bio-Rad, USA) 

### 4.13. RNA Isolation and Reverse Transcription-Polymerase Chain Reaction (RT-PCR) Analysis 

After treated the cells with different concentrations of DEN or DEN-HPβCD, Total RNA was isolated by using a Trizol RNA isolation kit (Invitrogen, Carlsbad, CA, USA) and stored at −80 °C for subsequent analysis. First-strand cDNA was synthesized from 600 ng of RNA by using iScript cDNA Synthesis kit (Bio-Rad). MyTaq Mix kit (Bioline, Taunton, MA, USA) was used for amplification. The sequences of primers used in RT-PCR were shown in Table 1. The PCR cycling conditions for caspase-3 were 95 °C for 1 min and the amplification was followed by 30 cycles of denaturing at 95 °C for 15 s, annealing at 60 °C for 15 s, and primer extension a 72 °C for 10 s with a final extension at 72 °C for 7 min. The PCR cycling conditions for β-actin were 95 °C for 1min and the amplification was followed by 30 cycles of denaturing at 95 °C for 15 s, annealing at 61 °C for 15 s, and primer extension a 72 °C for 10 s with a final extension at 72 °C for 7 min. The PCR product was analysed on 1.5% agarose gels and DNA bands were visualized by 5× loading buffer and Bio rad gel documentation system/Bio-Rad. All experiments were performed in triplicate.

### 4.14. Statistical Analysis

All the experiments were concluded in triplicate, and the results were reported in terms of mean ± SD. According to the SPSS, statistical analysis was accomplished through the experiments. The analysis of variance was carried out using the ANOVA technique, and a value of *p* < 0.05 was deemed to be of statistical significance.

## 5. Conclusions

In conclusion, this study was able to establish that DEN-HPβCD complex is a potent compound that is capable of inducing apoptosis in metastatic colon cancer in vitro. Apoptosis was achieved via multiple cancer-signaling pathways such as Fas mediated, intrinsic, ROS and cell cycle arrest. 

Further studies are needed to extrapolate the in vitro cytotoxic effects of the complex to in vivo anticancer application. More research is also needed to determine the half-life, stability, and the biologically significant concentrations needed to induce the apoptotic pathway in vivo. Therefore, our findings strongly suggest that the DEN-HPβCD complex deserves further investigation as a potential cancer chemotherapeutic agent.

## Figures and Tables

**Table 1:** The sequences of primers used in RT-PCR.

Primers	Forward primer (5′–3′)	Reverse primer (5′–3′)
capspase-3	TCA CAG CAA AAG GAG CAG TT	CGT CAA AGG AAA AGG ACT CA
β-actin	TCA CCC TGA AGT ACC CCA TC	CCA TCT CTT GCT GCA AGT CC
